# A Novel Parameter for Fatigue Damage Assessment of Laser-Repaired Nickel-Based Alloy

**DOI:** 10.3390/ma16010047

**Published:** 2022-12-21

**Authors:** Jianyu Zhao, Chuanqing Geng, Huimin Xie, Fei Liu

**Affiliations:** School of Aerospace Engineering, Tsinghua University, Beijing 100084, China

**Keywords:** damage parameter, fatigue damage, laser repairing, nickel-based alloy, strain standard deviation

## Abstract

The fatigue damage assessment of laser-repaired components is critical to their service safety. However, since laser repairing is an advanced green remanufacturing technology, the current research on its fatigue mechanical behavior and fatigue damage evaluation methods is still immature. In addition, the relevant models used for the fatigue damage evaluation can only indicate the fatigue limit of components, which cannot describe the damage accumulation process of the components during the fatigue testing. Therefore, there is an urgent need to develop a fatigue damage evaluation method that can describe the fatigue damage accumulation and evolution to reveal the damage evolution mechanism during the fatigue test. In this study, based on the 3D-DIC technique, new damage parameters, i.e., strain average value and strain standard deviation, are proposed to quantitatively describe the damage status of the nickel-based components during the stress-based fatigue process. Then, based on the new damage parameters, a strain average value/strain standard deviation damage curve method is proposed to describe the damage status evolution of the components during the fatigue testing and evaluate its fatigue damage. For example, in the tensile fatigue test, the strain average value/strain standard deviation damage curves of the substrate component and the laser-repaired component can be divided into two damage stages. In the first damage stage, the damage increases slowly with the increase in the cycle number, whereas in the second damage stage, the damage increases rapidly with the increase in the cycle number. At this time, there is a demarcation point between the first damage stage and second damage stage in the strain average value damage curve and strain standard deviation damage curve. The cycle number of the demarcation point can be used as a reference value for the fatigue failure of the laser-repaired component. In addition, the electron backscatter diffraction (EBSD) technique was used to verify the validity of the evaluation results from the novel damage parameters.

## 1. Introduction

Nickel-based alloys are commonly used superalloys which have been widely used in the manufacture of critical hot-section parts of aeroengines, such as blades [1,2]. However, under the condition of multi-physics coupling, the blades are very prone to fatigue-related damage or destruction [3]. Therefore, the remanufacturing of damaged blades, restoring their size and mechanical properties, is key to extending the service life of aeroengines. In recent years, with the development of laser repairing technology based on the principle of directed energy deposition (DED), the application of the laser repairing technology in the field of repairing the damaged components of aeroengines has drawn more and more attention [4,5,6,7,8,9]. However, numerous defects are formed during the laser repairing due to the processing characteristics of point-by-point deposition, which can greatly affect the fatigue properties of the laser-repaired component. Currently, the fatigue behavior of the substrate component has been systematically investigated while that of the laser-repaired component requires further study. Therefore, the method of evaluating the fatigue damage of the laser-repaired alloys is important for clarifying its fatigue failure mechanism.

At present, the methods for characterizing the fatigue behavior of the components mainly include stress/strain-based methods, fracture mechanics methods, and continuum damage mechanics (CDM) methods [10,11,12,13,14,15,16,17,18,19,20]. The stress/strain-based methods mainly include the stress-based life evaluation method, e.g., the Basquin equation and the strain-based life evaluation method, e.g., the Morrow method and the Manson–Coffin formula [12,13,14,15]. For instance, Whittaker et al. [11] used the Walker strain method to study the effects of temperature and stress ratios on fatigue damage parameters, and the results showed that the Walker strain equation can better evaluate the fatigue damage of the nickel-based components in the range of 600–750 °C. The fracture mechanics method is mainly based on Paris’ law [10,11,12,13,14,15,16]. The CDM-based methods are used to evaluate the fatigue damage of the component by calculating the damage parameters [17,18,19,20]. Kim et al. [17] developed a fatigue life model based on CDM for predicting the fatigue life of nickel-based alloys under creep–fatigue interactions. Lemaitre et al. [18] and Chaboche et al. [19] proposed strain equivalence hypotheses that combine the theory of CDM with the assessment of fatigue damage. In addition to the above evaluation methods for fatigue failure, the digital image correlation (DIC) method as a full-field deformation measuring technique is often used to characterize the fatigue crack propagation (FCP) behavior of the component [21,22,23,24,25,26,27,28]. For example, Mcneill et al. [21] used the displacement data calculated by the DIC method to determine the stress intensity factor $K_{I}$ of the components with different geometries. Sánchez et al. [22] reported that the fatigue crack growth (FCG) rate curve can be obtained using the DIC method combined with the finite element method (FEM), and then the fatigue damage of the EA1N steel can be evaluated. Hosdez et al. [25] compared the FCP law of cast iron obtained by the DIC method and direct current potential drop (DCPD) method, respectively. The results show that the two methods both have high accuracy in describing the FCP law, but the DIC method can also study the plasticity of the crack tip. In addition, Roux-Langlois et al. [26] proposed the unified DIC/extended FEM method based on the Williams’ series to identify the FCG law of pure titanium T35. However, although the above methods can well evaluate the fatigue damage of the component, the stress/strain-based methods can obtain the fatigue limit of the component, which cannot well reflect the continuous damage accumulation and evolution during the fatigue testing. In addition, the DIC method is often used to evaluate the FCP life of the component, whereas the reports about the fatigue damage evaluation of the component without pre-crack using the DIC method are relatively rare. Therefore, there is an urgent need to develop a fatigue damage evaluation method based on the DIC technique which can describe the continuous damage accumulation and evolution of the component without pre-crack during the fatigue testing.

In this paper, in view of the relevant requirements and based on the fatigue-photomechanical test system established earlier [29,30], we propose new damage parameters, the strain average value and strain standard deviation, to describe the evolution of the damage status of the nickel-based components during the fatigue testing and use the strain average value/strain standard deviation damage curves to evaluate the fatigue damage. In addition, the EBSD technique was used to confirm that the strain standard deviation is a damage parameter that reflects the degree of local strain concentration.

## 2. Experimental Procedures

### 2.1. Experimental Materials and Laser Repairing Process

**Figure 1:**
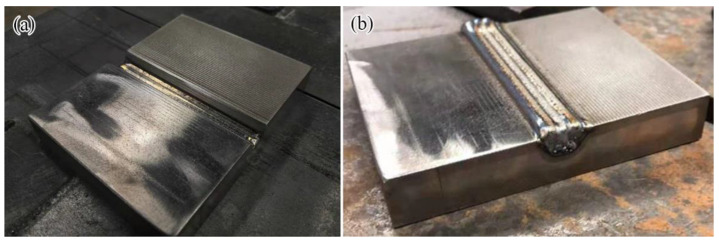
Laser-repaired nickel-based components: (**a**) before laser repairing; (**b**) after laser repairing.

The substrate material used in this study was GH4169 bulk alloy, and its chemical composition is given in Table 1. The substrate component with dimensions of 90 × 70 × 15 mm^3^ were machined into the component containing the body-shaped groove notch to be repaired (Figure 1a). The bottom width of the body-shaped groove notch was 6 mm, the top width was 10 mm, the length was 70 mm, and the depth was 5 mm. Then, the body-shaped notch of the substrate component was repaired and shaped using the laser DED technique (Figure 1b). The chemical composition of the repairing powder is given in Table 1.

Before laser repairing, the substrate component with the notch was polished with sandpaper and cleaned with absolute ethanol to ensure the alloy had the cleanliness and roughness required by the laser repairing experiment, as shown in Figure 1a. The coaxial powder feeding method for DED fabrication was adopted, and the laser processing system (LDF3000–60VGP, supplied by Laserline GmbH, Mülheim-Kärlich, Germany) with laser wavelength of 1064 nm was used for the laser repairing experiment. The laser repairing parameters are given in Table 2.

### 2.2. BSL 3D-DIC Measurement System

**Figure 2:**
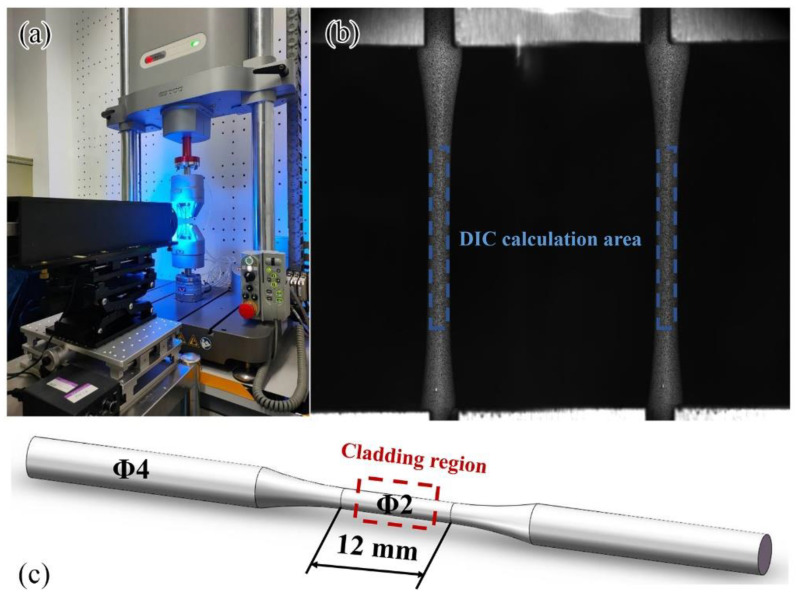
Deformation measurement system: (**a**) BSL 3D-DIC measurement system and fatigue testing machine; (**b**) DIC calculation area (90 × 850 Pixel); (**c**) fatigue component size, the laser cladding region (10 × 2 × 2 mm^3^) is shown schematically in the red square.

The digital image correlation (DIC) method is a measurement technology based on image analysis and feature matching which uses the surface image of the component before deformation as the reference image, and the correlation analysis is carried out with the image after deformation to obtain the full-field displacement of the component surface and then obtains the strain field [30,31]. In this study, the integrated three-dimensional digital image correlation system with biprism-based single lens (BSL 3D-DIC) was used to measure the full-field deformation of the component surface [32]. The BSL 3D-DIC system is composed of a charge-coupled device (CCD) camera, bilateral telecentric lens, and biprism. In order to avoid color crosstalk of the lights with different wavelengths, a blue-light-emitting diode (LED) light source is fixed at the front end of the measurement system, and blue-light illumination is used to measure strain field, as shown in Figure 2a. The BSL 3D-DIC deformation field measurement system has a maximum measurement accuracy of 0.01 pixel, a theoretical displacement measurement accuracy of 0.17 μm, a vertical strain measurement accuracy of 5 με, and a horizontal strain measurement accuracy of 10 με. The DIC calculation area is shown in Figure 2b.

### 2.3. Tensile and Fatigue Testing

**Figure 3:**
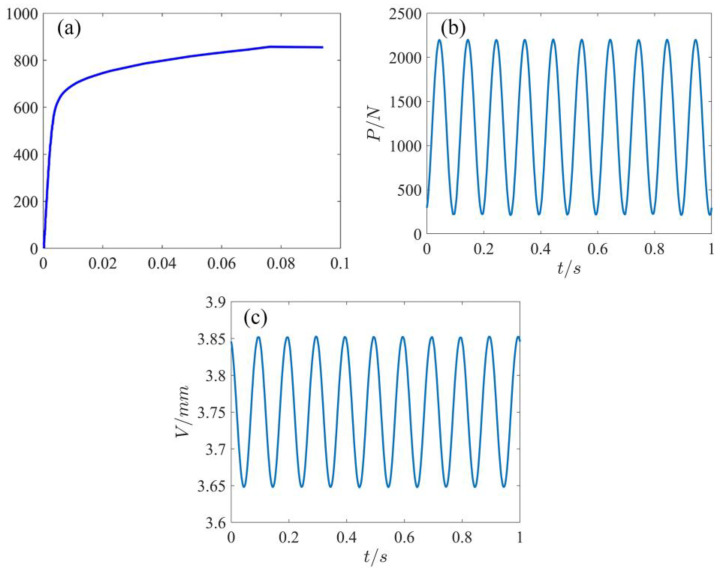
Strain–stress curve during tensile test and load–time curve and displacement–time curve during fatigue test: (**a**) strain–stress curve; (**b**) load–time curve; (**c**) displacement–time curve.

The round-bar-type component was used for tensile and fatigue tests, and the component size is shown in Figure 2c. The universal testing machine (Zwick Z020, supplied by ZwickRoell GmbH & Co. KG, Ulm, Germany) was used for tensile testing with a tensile loading rate of 0.1 mm/min. During the loading process, the BSL 3D-DIC system was used to record the component speckle field and analyze the full-field deformation of the component surface. The area from the center to both sides of the gauge length section of the specimen included the repaired area (the center area of the gauge length section), the repaired boundary, and the substrate area (both sides of the gauge length section). The mechanical properties data of the specimen containing the repaired area are given in Table 3, and its strain–stress curve is shown in Figure 3a. Because the surface of the component was relatively smooth after machining, it was impossible to directly distinguish the repaired area and the substrate area (Figure 1c). Therefore, the EBSD technique was used to characterize the repaired area in the center of the gauge length section of the component.

The component was cyclically loaded using a fatigue testing machine (Instron E3000, supplied by Instron, Norwood, MA, USA). The waveform was sine, loading frequency was 10 Hz, and stress ratio was 0.1. In the fatigue experiment, speckle images of the fatigue component’s surface were collected using the BSL 3D-DIC system to calculate the axial (loading direction), transverse (perpendicular to the loading direction), and tangential (shear direction) deformation fields of the components. The load–time curve and displacement–time curve during the fatigue test are shown in Figure 3b,c.

### 2.4. Microstructure Characterization

EBSD images of the fatigue components were acquired using the high-resolution and high-sensitivity surface analysis system (S9000X, supplied by Carl Zeiss AG, Oberkochen, Germany) with an acceleration voltage of 20 kV. EBSD specimens were prepared by electropolishing technique. The electrolyte was perchloric acid and ethanol solution with a volume ratio of 1:9. The electropolishing voltage was 20 V and the time was 10–20 s. The fatigue fracture morphology of the repaired component was observed with the scanning electron microscope (SS550 SEM, supplied by SHIMADZU corporation, Tokyo, Japan).

### 2.5. New Damage Parameters Based on Statistical Analysis of DIC Strain Field

In order to evaluate the fatigue damage of components efficiently and simply, the new damage parameters, strain average value and strain standard deviation, were proposed. Based on the BSL 3D-DIC measurement results, the average value of axial strain (tensile direction) damage curve and standard deviation of axial strain damage curve of the substrate component and laser-repaired component were drawn to quantitatively characterize the fatigue damage behavior.

## 3. Experimental Results and Discussion

### 3.1. Deformation Mechanism of Substrate and Laser-Repaired Nickel-Based Components during Tensile Testing

**Figure 4:**
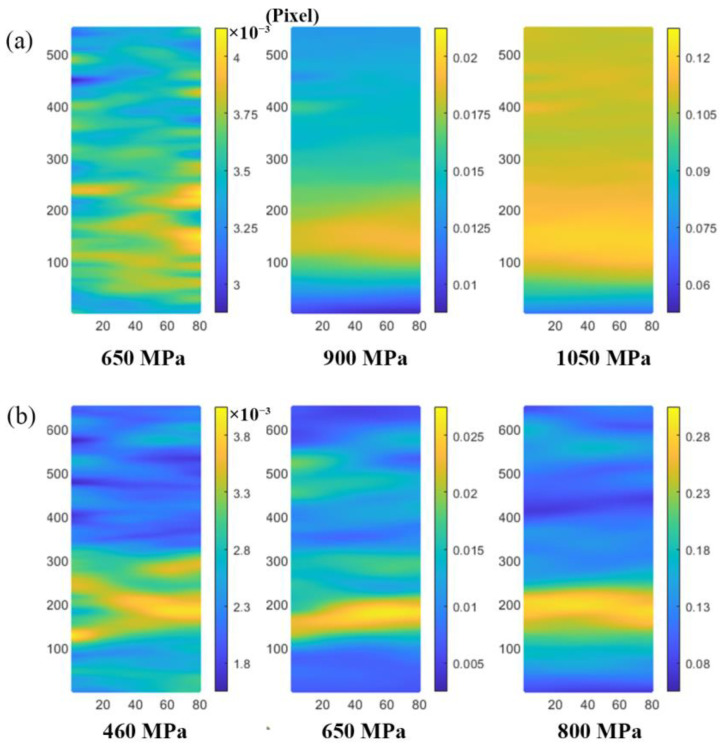
Axial strain fields of substrate component and laser-repaired component under different tensile loads, the area size of the images for DIC calculation is 90 × 850 Pixel: (**a**) substrate component, 650 MPa corresponding to elastic stage, 900 MPa corresponding to plastic stage, 1050 MPa corresponding to before fracture failure; (**b**) laser-repaired component, 460 MPa corresponding to elastic stage, 650 MPa corresponding to plastic stage, 800 MPa corresponding to before fracture failure.

Figure 4 shows the strain field distribution maps of the substrate component and laser-repaired component at the elastic stage, plastic stage, and before fracture failure during the tensile testing. Compared with the substrate component, the repaired component is prone to produce a strain concentration during the tensile testing, and the degree of strain concentration increases significantly with the increase in the tensile load. As shown in Figure 4b, there are many areas with a high strain concentration on the surface of the repaired component during the elastic deformation stage. With the increase in the tensile load, due to the flow of the metal, the strain is concentrated towards a certain region (650 MPa), further increasing the tensile load, and the strain concentration in this strain concentration area increases significantly (800 MPa) until fracture failure occurs. As shown in Figure 4a, multiple strain concentration areas (650 MPa) are formed on the substrate component surface in the elastic stage. With the increase in the tensile load, the strain concentration areas gradually increase. Before fracture failure, the strain is uniformly distributed in the substrate component (1050 MPa). As a result of the formation of the repaired interface in the laser-repaired component, the damage is preferentially accumulated at the repaired interface [29,30], which aggravates the deformation heterogeneity of the repaired component, resulting in the strength of the repaired component being lower than that of the substrate component.

**Figure 5:**
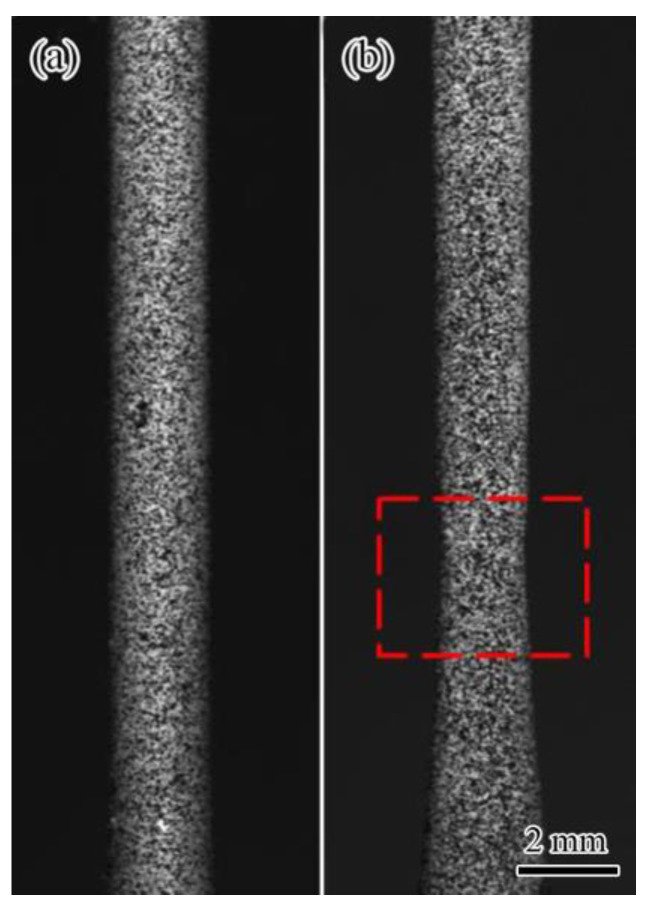
Macroscopic plastic deformation of nickel-based component during tensile process: (**a**) substrate component; (**b**) laser-repaired component, necking is shown in red square area.

The morphologies of the macroscopic plastic deformation of the substrate component and repaired component during the tensile testing are shown in Figure 5. There is no obvious macroscopic deformation in the substrate component during the tensile process, whereas the repaired component produces obvious necking, indicating that the strain is concentrated in the local area of the repaired component, resulting in strong heterogeneous plastic deformation. The reduction in the area of the substrate component after fracture is only 20%, whereas that of the repaired component is as high as 51%. In conclusion, in the process of the uniaxial tension, the deformation mechanism of the substrate component is homogeneous deformation, whereas that of the laser-repaired component is heterogeneous deformation.

### 3.2. Strain Field Evolution and Fracture Mechanism of Nickel-Based Components during Fatigue Process

**Figure 6:**
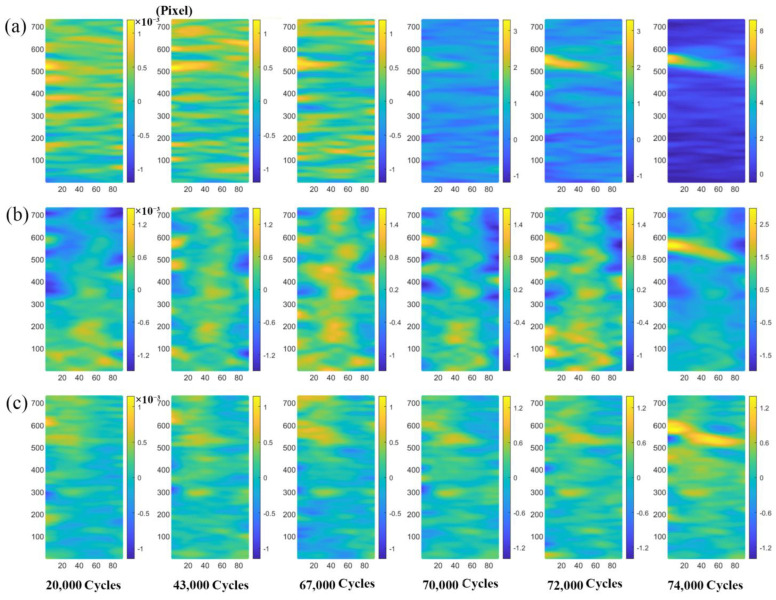
Strain fields of substrate component in different directions under different cycles, the area size of the images for DIC calculation is 90 × 850 Pixel: (**a**) axial strain field; (**b**) transverse strain field; (**c**) tangential strain field.

Figure 6 shows the measured results of the axial, transverse, and tangential strain fields of the substrate component under a fatigue load of 800 MPa and different cycles. It can be seen from Figure 6 that the fatigue damage process can be divided into two stages: the first damage stage is when the cycle number is less than 70,000 cycles. In this stage, the strain field distribution in three deformation directions is relatively uniform, and no area with a high degree of local strain concentration is formed. When the cycle number is more than 70,000 cycles, the substrate component enters the second damage stage, that is, the degree of local strain concentration increases significantly with the increase in the cycle number. With the increase in cycles from 72,000 to 74,000, the axial strain amplitude significantly increases from 2500 με to 90,000 με, and transverse strain amplitude increases from 2000 με to 35,000 με, whereas the tangential strain amplitude increases from 1000 με to 10,000 με. In conclusion, during the fatigue loading process, the degree of strain concentration in different directions of the substrate component is different, and the magnitude of the degree of the strain concentration is: axial strain > transverse strain > tangential strain. Since the axial strain of the substrate component changes significantly, this paper focuses on the evolution of the axial strain field of the substrate component and laser-repaired component.

**Figure 7:**
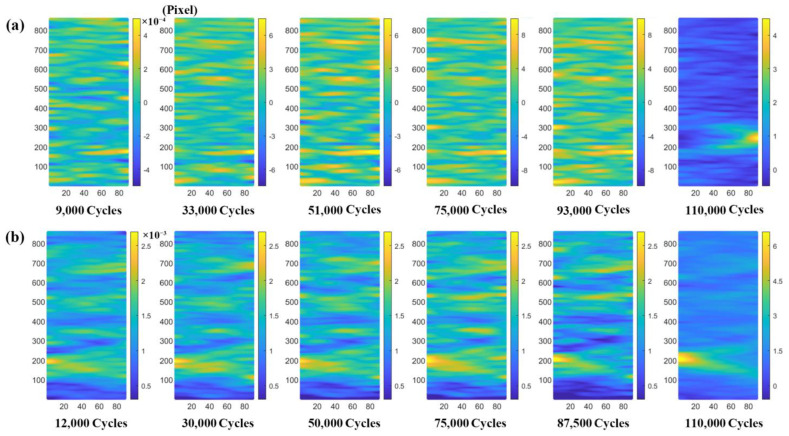
Axial strain field of laser-repaired component under different loads and cycles, the area size of the images for DIC calculation is 90 × 850 Pixel: (**a**) 680 MPa; (**b**) 700 MPa.

Figure 7 shows the distribution maps of the axial strain field of the laser-repaired component under different fatigue loads. Due to the different fatigue loads applied, the cycle numbers of the two types of components (substate and repaired) entering the first damage stage and the second damage stage are different. Therefore, in order to obtain the strain field distribution maps of the first damage stage and the second damage stage of the two types of components, the component images were collected at different cycle numbers. The comparison of Figure 7 shows that under a certain number of cycles, with the increase in the cycle number, the degree of strain concentration under different fatigue loads slowly increases, and the distribution of the strain field does not change significantly. Similar to the substrate component, when the cycle number exceeds a certain critical value, the strain concentration degree of both components increases significantly, and the strain concentration occurs in the local area, leading to fatigue failure. Studies have shown that the residual strain of the laser-repaired nickel-based component mainly exists in the form of local concentration [29,30], which is consistent with our experimental results. According to the analysis in Figure 6 and Figure 7, when strong local strain concentration occurs, fatigue failure will occur. Therefore, the fatigue damage of the tested component can be accurately evaluated by quantitatively characterizing the relationship between the degree of strain concentration and the number of cycles in the fatigue process. Based on the above analysis, the new damage parameters, strain average value and strain standard deviation, are proposed to evaluate the fatigue damage of the components.

**Figure 8:**
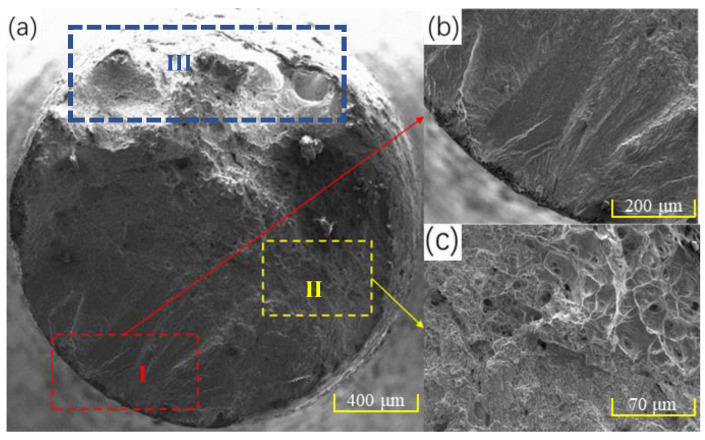
Fatigue fracture morphology of laser-repaired component under 700 MPa: (**a**) fatigue fracture morphology; (**b**) crack source region; (**c**) crack propagation region. (The crack source region, crack propagation region, and transient fracture region are shown in I, II, and III areas in Figure 8a, respectively).

Figure 8 shows the fatigue fracture morphology of the laser-repaired component when the fatigue load is 700 MPa. The fatigue fracture morphology can be divided into three regions, namely, crack source region (I), crack propagation region (II), and transient fracture region (III). As shown in Figure 8, the fatigue cracks form on the surface of the repaired component and then propagate into the interior of the component. A large number of dimples with different sizes have been formed in the crack propagation region, indicating that the microstructure has a strong heterogeneity. With the further increase in cycle numbers, fatigue fracture occurs in the laser-repaired component, as shown in region III in Figure 8a. Since a large number of the dimples are formed after fatigue fracture, the fatigue fracture mechanism is ductile fracture.

### 3.3. Fatigue Failure Analysis Based on New Damage Parameters

**Figure 9:**
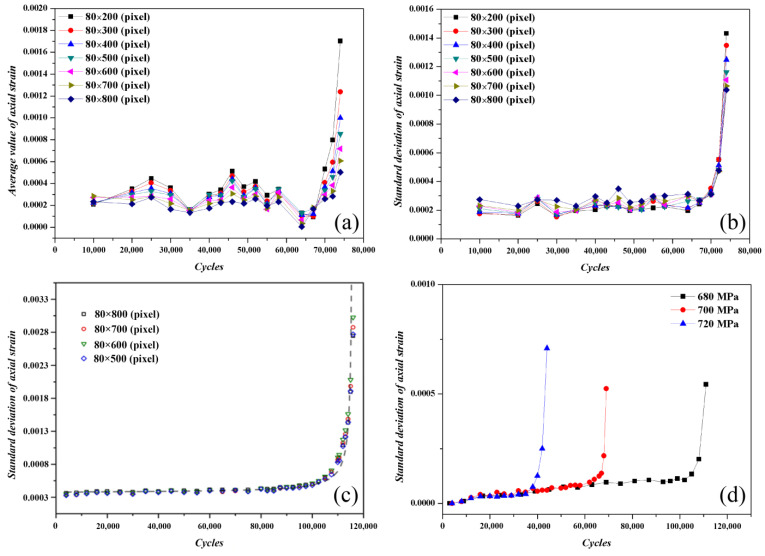
Strain average value damage curve and strain standard deviation damage curve of substrate component and laser-repaired component: (**a**) strain average value damage curves of substrate component with different DIC-calculated subset sizes; (**b**) strain standard deviation damage curves of substrate component with different DIC-calculated subset sizes; (**c**) strain standard deviation damage curves of laser-repaired component with different DIC-calculated subset sizes; (**d**) strain standard deviation damage curve of laser-repaired component under different cyclic loads.

Figure 9 shows the average value of axial strain damage curve and the standard deviation of axial strain damage curve of the substrate component (Figure 9a,b) and the laser-repaired component (Figure 9c,d) based on the results of the DIC strain fields in Figure 6 and Figure 7, respectively. As shown in Figure 9, the damage process of the substrate component and laser-repaired component includes two stages. In the first damage stage, the strain average value and strain standard deviation remain stable with the increase in fatigue cycles and fluctuate in a small range. When the damage accumulates to a certain extent, the strain average value and strain standard deviation will suddenly increase, that is, demarcation points appear between the first damage stage and second damage stage in the strain average value damage curve and strain standard deviation damage curve. After the demarcation point appears, fatigue fracture will occur with the further increase in the cycle numbers. At this time, the demarcation point in the curve can be regarded as the critical point of fatigue damage accumulation, and therefore the strain average value and the strain standard deviation can be used to quantitatively characterize the fatigue damage degree. According to the above analysis, when the fatigue cycle does not exceed the cycle number corresponding to the demarcation point in the strain average value damage curve or strain standard deviation damage curve, the component is safe in service.

In addition, the influence of the size of the DIC-calculated subset on the evolution of the strain average value damage curve and the strain standard deviation damage curve was evaluated in the same substrate component. In this study, the axial direction of the round-bar-type component is the length direction, and the transverse direction is the width direction. Rectangular areas with a width of 80 pixels and a length of 200–800 pixels were selected for study. The experimental results are shown in Figure 9a,b, and the DIC calculation area of the components is shown in Figure 2b. As shown in Figure 9a,b, the calculated subset size of the substrate component has almost no influence on the shape of the curve (the evolution law of the curve) but affects the values of the strain average value and the strain standard deviation. The larger the calculated subset size, the greater the fluctuation in the strain average value and the strain standard deviation. The fluctuation in the strain average value and the strain standard deviation is mainly related to the strain heterogeneity. The larger the calculated subset size, the greater the number and density of the second-phase particles, dislocations, and other microstructures in the area. Due to the heterogeneous distribution of the second-phase particles, the deformation of the substrate component in different areas is heterogeneous. In addition, because the interaction between the second-phase particles and dislocations in different regions of the substrate component is different, this also leads to heterogeneous deformation, which increases the fluctuation in the strain average value and the strain standard deviation of the substrate component.

**Figure 10:**
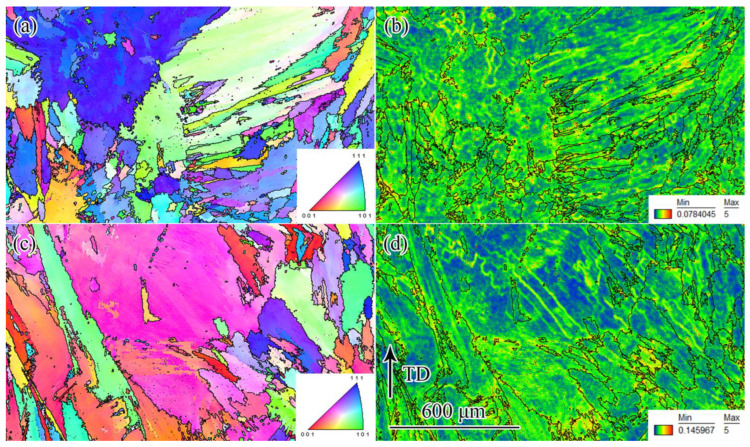
IPF figures and KAM maps of laser-repaired components under different cyclic loads; TD is the tensile direction, IPF is inverse pole figure, KAM is kernel average misorientation: (**a**,**b**) 700 MPa; (**c**,**d**) 720 MPa.

It can be seen from the above analysis that the larger the calculated subset size, the greater the fluctuation in the strain average value and the strain standard deviation of the substrate component. The comparison result of Figure 9a–c shows that the effect of the calculated subset size on the fluctuation in the strain standard deviation is relatively small, which indicates that using the strain standard deviation damage curve to evaluate the fatigue damage of the alloy has good robustness. A relatively large rectangular area with a width of 80 pixels and a length of 500–800 pixels was selected to study the influence of the calculated subset size on the strain standard deviation of the laser-repaired component, as shown in Figure 9c. The effect of the calculated subset size on the strain standard deviation is not obvious, and the strain standard deviation damage curve can accurately reflect the damage accumulation and fatigue failure process during the fatigue testing. In fact, the slope of the strain standard deviation damage curve is approximately zero at the first damage stage, indicating that the damage accumulation rate of the alloy is lower, whereas the slope of the curve is approximately infinite at the second damage stage, indicating that the damage accumulation rate is higher, i.e., the laser-repaired component is in an accelerated failure state. The fatigue damage of the laser-repaired components under different fatigue loads was evaluated using the strain standard deviation damage curve, as shown in Figure 9d. The cyclic load value greatly affects the fatigue damage of the laser-repaired component, and the larger the applied cyclic load, the shorter the fatigue life. When the cyclic load is 680 MPa, the fatigue life reaches 110,000 cycles, whereas when the cyclic load is increased to 720 MPa, the fatigue life is significantly shortened to only 40,000 cycles. In addition, when the cyclic load is 720 MPa, the strain standard deviation is the largest, whereas when the cyclic load is 700 MPa and 680 MPa, the strain standard deviation is almost equal. We hypothesized that the strain standard deviation should reflect the degree of strain concentration in the local area of the component, and the greater the strain standard deviation, the greater the degree of local strain concentration, that is, the greater the deformation heterogeneity of the component. In order to prove the above point of view, EBSD characterization was performed on the post-fatigue repaired component under cyclic loads of 700 MPa and 720 MPa to evaluate the residual deformation field, as shown in Figure 10. There is no obvious difference in the microstructure of the laser-repaired components under different cyclic loads after fatigue, and their grain structures are coarse columnar grains, lamellar grains, and fine equiaxed grains (Figure 10a,c). The comparison of Figure 10b,d shows that when the cyclic load increases from 700 MPa to 720 MPa, the heterogeneity of the strain field distribution is significantly enhanced, that is, the local strain concentration is significantly increased, which is consistent with the results in Figure 9d. In addition, when the cyclic load is 700 MPa, the minimum KAM value of the repaired component is 0.0784045, whereas when the cyclic load increases to 720 MPa, the minimum KAM value reaches 0.145967, indicating that with the increase in the cyclic load, the strain concentration increases significantly, which indicates that the strain standard deviation is a damage parameter reflecting the strain concentration degree in the local area after fatigue. Therefore, the larger the strain standard deviation, the greater the degree of strain concentration in the local area, which promotes fatigue damage of the component.

## 4. Conclusions

Based on the BSL 3D DIC technique, this paper proposes new damage parameters to quantitatively characterize the fatigue damage of the nickel-based components, and these parameters can not only evaluate the fatigue damage degree but also describe the continuous evolution of the fatigue damage during the stress-based fatigue process. In addition, the EBSD technique was used to verify the validity of the new damage parameters to characterize the damage degree of the tested components, and the following research conclusions can be drawn:

(1) Based on the BSL 3D-DIC technique, a method to evaluate the fatigue damage of the substrate component and laser-repaired component was proposed by using the strain average value/strain standard deviation damage curves. The strain average value/strain standard deviation damage curves can be divided into two damage stages. In the first damage stage, the damage degree slowly increases with the increase in the cycle number, whereas in the second damage stage, the damage degree sharply increases with the increase in the cycle number. The effect of the subset size (DIC calculation) on the fluctuation in the strain standard deviation damage curve is not obvious compared with the strain average value damage curve, which indicates that the strain standard deviation damage curve should be suitable for the evaluation of fatigue damage of the components.

(2) There is a demarcation point between the first damage stage and second damage stage in the strain average value damage curve and strain standard deviation damage curve. The cycle number corresponding to this demarcation point can be used as a reference value for the damage safety design of the substrate component and the laser-repaired component.

(3) The strain average value reflects the full-field damage degree of the component. EBSD results show that with the increase in the strain standard deviation, the KAM value and local strain concentration of the laser-repaired component increase, which indicates that the strain standard deviation reflects the damage degree of the local area.

## Figures and Tables

**Table 1:** Chemical compositions of substrate material and repairing powder (wt%).

Element	Ni	Fe	Cr	Nb	Mo	Ti	Al	Mn
Substrate Powder	50.34 51.08	20.74 13.87	18.44 19.24	5.50 5.28	3.27 3.13	0.80 0.87	0.50 0.60	0.40 0.05

**Table 2:** Laser repairing parameters.

Laser Power (W)	Scanning Rate (mm/s)	Laser Beam Diameter (mm)
1100	5	3

**Table 3:** Mechanical properties data of the specimen containing repaired area.

Young’s Modulus (GPa)	Yield Strength (MPa)	Ultimate Tensile Strength (MPa)	Strain-Hardening Exponent (*n*)	Strength Coefficient (*K*)
176	633	856	0.1025	525.8

## Data Availability

The data presented in this study are available upon request from the corresponding author.

## Nomenclature

$\sigma_{0.2}$	Yield strength, MPa
$\sigma_{b}$	Ultimate tensile strength, MPa
*E*	Young’s modulus
*K*	Strength coefficient
*n*	Strain-hardening exponent
BSL	Biprism-based single lens
CCD	Charge-coupled device
CDM	Continuum damage mechanics
DCPD	Direct current potential drop
DED	Directed energy deposition
DIC	Digital image correlation
EBSD	Electron backscatter diffraction
FCG	Fatigue crack growth
FCP	Fatigue crack propagation
FEM	Finite element method
KAM	Kernel average misorientation
LED	Light-emitting diode
SEM	Scanning electron microscope
TD	Tensile direction
